# Vaccination of Elk (*Cervus canadensis*) with *Brucella abortus* Strain RB51 Overexpressing Superoxide Dismutase and Glycosyltransferase Genes Does Not Induce Adequate Protection against Experimental *Brucella abortus* Challenge

**DOI:** 10.3389/fcimb.2016.00010

**Published:** 2016-02-10

**Authors:** Pauline Nol, Steven C. Olsen, Jack C. Rhyan, Nammalwar Sriranganathan, Matthew P. McCollum, Steven G. Hennager, Alana A. Pavuk, Phillip J. Sprino, Stephen M. Boyle, Randall J. Berrier, Mo D. Salman

**Affiliations:** ^1^Wildlife Livestock Disease Investigations Team, Veterinary Services, Animal and Plant Health Inspection Services, United States Department of AgricultureFort Collins, CO, USA; ^2^Agricultural Research Services, United States Department of Agriculture, National Animal Disease CenterAmes, IA, USA; ^3^Center for Molecular Medicine and Infectious Diseases, Virginia Maryland College of Veterinary Medicine, Virginia Polytechnic Institute and State UniversityBlacksburg, VA, USA; ^4^National Veterinary Services Laboratories, Veterinary Services, Animal and Plant Health Inspection Services, United States Department of AgricultureAmes, IA, USA; ^5^Department of Pathobiology and Microbiology, College of Veterinary Medicine and Biological Sciences, Colorado State UniversityFort Collins, CO, USA; ^6^Colorado Serum CompanyDenver, CO, USA; ^7^Department of Clinical Sciences, College of Veterinary Medicine and Biological Sciences, Colorado State UniversityFort Collins, CO, USA

**Keywords:** *Brucella abortus*, RB51, elk, wildlife vaccination, superoxide dismutase, O-side chain, glycosyltransferase

## Abstract

In recent years, elk (*Cervus canadensis*) have been implicated as the source of *Brucella abortus* infection for numerous cattle herds in the Greater Yellowstone Area. In the face of environmental and ecological changes on the landscape, the range of infected elk is expanding. Consequently, the development of effective disease management strategies for wild elk herds is of utmost importance, not only for the prevention of reintroduction of brucellosis to cattle, but also for the overall health of the Greater Yellowstone Area elk populations. In two studies, we evaluated the efficacy of *B. abortus* strain RB51 over-expressing superoxide dismutase and glycosyltransferase for protecting elk from infection and disease caused by *B. abortus* after experimental infection with a virulent *B. abortus* strain. Our data indicate that the recombinant vaccine does not protect elk against brucellosis. Further, work is needed for development of an effective brucellosis vaccine for use in elk.

## Introduction

The persistence of brucellosis in wild elk (*Cervus canadensis*) remains a serious concern in the Greater Yellowstone Area (GYA). In recent years, elk have been implicated as the source of infection for numerous cattle herds in Wyoming, Idaho, and Montana, USA (Hillman, 2002; Olsen et al., 2006; Rhyan et al., 2013). Consequently, the development of effective disease management strategies for wild elk herds is of utmost importance, not only for the prevention of reintroduction of brucellosis to cattle, but also for the overall health of the GYA elk populations. A valuable component of brucellosis management in wildlife would be utilization of an effective vaccine that could be delivered mucosally. Currently available commercial vaccines for use in cattle (*B. abortus* strains 19 [s19] and RB51 [sRB51]) have offered little to no protection in elk against *Brucella*-induced abortion and infection (Cook et al., 2002; Kreeger et al., 2002; Olsen et al., 2002). Vemulapalli et al. (2000a,b, 2002) have shown that recombinant *B. abortus* sRB51 over-expressing antigenic factors in the form of either homologous Cu/Zn superoxide dismutase (*SOD*c) or glycosyltransferase (encoded by *wbo*A; McQuiston et al., 1999) intra-cytoplasmically (sRB51+*SOD*c, *Wbo*A), induces an enhanced immune response and thus greater protection against *B. abortus* challenge in mice as compared to vaccination with sRB51 alone.

Strain RB51, a rough mutant of *B. abortus*, does not express O-side chain, a component of the bacterium's lipopolysaccharide (LPS), which is essential for the structure and function of the outer membrane of Gram-negative bacteria (Cardoso et al., 2006). Glycosyltransferase is vital for O-side chain biosynthesis of the LPS in *Brucella* spp. Additionally, it is the host antibody response against the O-side chain that is detected by standard *Brucella* serologic tests. *Brucella* LPS has been shown to impair antimicrobial host responses (reviewed by Seleem et al., 2008). Although, the LPS of *Brucella* species are less toxic than LPSs of most other species of Gram-negative bacteria (Forestier et al., 1999, 2000; Lapaque et al., 2005), studies have demonstrated that *Brucella* LPS inhibits complement, antibacterial-peptide activity, and host cell apoptosis, and prevents production of immune mediators (Forestier et al., 1999, 2000; Lapaque et al., 2005). These aspects allow *Brucella* species to evade destruction and persist within phagocytic cells. Also within the context of avoiding destruction, superoxide dismutases have been demonstrated to influence the oxidative environment of the host tissue and also play a role in intracellular survival of *Brucella* (Break et al., 2012; Wareth et al., 2015). Superoxide dismutases catalyze the dismutation of oxygen radicals, thus preventing the effects of reactive oxygen toxicity.

Our objectives were to investigate the efficacy of sRB51+*SOD*c, *Wbo*A in protecting elk against experimental challenge with virulent *B. abortus* strain 2308 (s2308). This investigation involved two separate controlled animal trials. Our results indicated that sRB51+*SOD*c, *Wbo*A would not be effective in protecting elk from *Brucella abortus* infection. Further, work is essential to develop a protective vaccine to manage brucellosis in wild elk in the GYA.

## Materials and methods

### Animals

#### Experiment 1

Twenty-one captive-raised adult female elk were obtained in July, 2005 and housed at the United States Department of Agriculture (USDA)/Colorado State University (CSU)-Animal Population Health Institute outdoor wildlife research facility (WRF) in Fort Collins, Colorado, USA. Elk were purchased from a commercial herd in Minnesota, USA. This herd was certified free of brucellosis, bovine tuberculosis, and chronic wasting disease. The animals were acclimated for 6 wk at the WRF prior to vaccination. Fifteen elk were vaccinated intramuscularly (prime) with sRB51+*SOD*c, *Wbo*A as described below in September 2005; and a subset of eight animals was orally boostered 6 wk after prime vaccination. Six animals served as non-vaccinated controls. These animals were exposed to bulls during the fall 2005 breeding season. In January 2006, elk were physically examined by rectal palpation for pregnancy. Six pregnant animals of each vaccine group were randomly selected for transportation in January, 2006 to a large animal Biosafety Level-3 (ABSL-3) facility for *B. abortus* s2308 challenge at the USDA, Agricultural Research Service, National Animal Disease Center (NADC), Ames, Iowa, USA. Only three elk in the control group were pregnant and were sent to the ABSL-3 facility. Three additional pregnant elk were obtained from the same commercial breeder and transported directly to the ABSL-3 facility. Animals were maintained under ABSL-3 conditions until the conclusion of the study in June, 2006.

#### Experiment 2

Twenty-nine adult female elk were obtained in January, 2008 from the wild in northeastern Oregon, USA, an area free of chronic wasting disease, and brought to the WRF. Animals were acclimated at the WRF for 2 mo before prime vaccination in March of 2008. Fourteen animals were orally vaccinated with sRB51+*SOD*c, *Wbo*A. Fifteen animals served as controls. Three animals in the control group died or were euthanized within the first 5 months of the study as a result of injuries or dystocia. Animals in the vaccinated group received booster vaccinations in December 2008. Elk were exposed to bulls during the 2009 fall breeding season. In December 2009, elk were examined for pregnancy status using ultrasound (Ibex, E. I. Medical Imaging, Loveland, Colorado, USA). Seven control cows and 13 vaccinates were diagnosed as pregnant. Due to space limitations, 11 randomly selected pregnant vaccinates and all seven control elk were transported to the ABSL-3 facility in February 2010 for experimental challenge. One vaccinate was ultimately determined not to be pregnant at necropsy.

All animals in both experiments were housed, handled, and cared for under protocols approved by the Institutional Animal Care and Use Committees of CSU and NADC.

### Vaccine preparation and vaccination

#### Experiment 1

*Brucella abortus* sRB51+*SOD*c, *Wbo*A was prepared for prime vaccination at Virginia Polytechnic Institute and State University (VaTech), Blacksburg, VA, USA, by growth on trypticase soy agar plates for 48 hr at 37°C and 5% CO2. The cells were harvested in phosphate buffered saline (PBS; pH 7.4), concentrated by centrifugation, washed twice in PBS and re-suspended in PBS. Vaccine concentration was determined by standard plate counts. The vaccine was then shipped overnight on ice to CSU, Fort Collins, CO where it was stored at 4°C for 24 hr before use.

For booster vaccination, sRB51 +*SOD*c, *Wbo*A was commercially prepared in lyophilized form (Colorado Serum Company (CSC), Denver, Colorado, USA) and reconstituted and used in accordance with manufacturer's recommendations.

Twenty-one elk were randomly divided into three groups. The first group (single dose; *n* = 7) received one intramuscular (IM) injection in the left hip with 1 ml of approximately 2 × 10^10^ colony forming units (cfu) of sRB51+*SOD*c, *Wbo*A (VaTech). The second group (oral boost; *n* = 8): received one IM injection in the left hip with 1 ml of approximately 2 × 10^10^ cfu sRB51+*SOD*c, *Wbo*A (VaTech) and was orally boosted 6 wk later with 1.2 ml of approximately 1 × 10^11^ cfu sRB51+*SOD*c, *Wbo*A (CSC). Oral inoculations were administered using an 8 French 16 inch urinary catheter inserted to the back of the pharynx via a cylindrical oral speculum. The third group (Control; *n* = 6) received 1 ml PBS IM in the left hip and served as controls. Controls were housed separately from vaccinated animals to prevent exposure to vaccine. Elk were transported to the ABSL-3 facility five mo post-prime vaccination and allowed to acclimate for 4 wk prior to *B. abortus* challenge.

#### Experiment 2

Twenty-nine captured elk were randomly divided up into two groups. In March 2008 one group (*n* = 14) was orally inoculated with a 5 ml volume of approximately 1 × 10^11^ cfu sRB51+*SOD*c, *Wbo*A prepared and provided by CSC, and orally boosted in a similar manner in December 2008. The second group was orally administered 5 ml diluent (CSC) (Control; *n* = 15). Controls were housed in a separate pen from vaccinated animals to prevent exposure to vaccine. Elk were transported to the ABSL-3 facility 22 mo post-prime vaccination and allowed to acclimate for 2 wk prior to *B. abortus* challenge.

### *Brucella abortus* preparation and challenge

For *Brucella* challenge in both experiments, smooth *B. abortus* s2308 was grown on tryptose agar containing 5% bovine serum for 48 hr at 37°C and 5% CO_2_. Bacteria were harvested by aspiration using saline. Suspensions of s2308 were adjusted to approximately 1 × 10^7^ cfu/ml by use of a spectrophotometer (wavelength-600 nm) and concentrations of viable bacteria were determined by standard plate counts.

At approximately mid-gestation in both experiments, animals were anesthetized with xylazine (0.6–0.8 mg/kg) and ketamine hydrochloride (2.5–3.0 mg/kg) IM and conjunctivally challenged with 1 × 10^7^ cfu s2308 (50 μl/eye).

### Sample collection and processing

#### Blood collection

Blood was collected via jugular venipuncture for serology prior to vaccination in both experiments; at 1, 2, 4, 6 (boost), 8, 12, 17, 22, and 30 wk after initial vaccination in Experiment 1; and at 2, 4, 8, 12, 14, 17, 21, 27 wk after initial vaccination in Experiment 2. In Experiment 1, at 6 and 10 wk post-prime vaccination, blood samples were collected for lymphocyte blastogenesis and flow cytometry. At 6 and 14 wk after experimental challenge, blood was collected for serology, lymphocyte blastogenesis, and flow cytometry as described earlier. In Experiment 2, post-challenge blood was obtained at 4, 8, and 12 wk for serology.

For both experiments, blood for serology was allowed to clot for 2–4 hr at 4°C, centrifuged, divided into 1-ml aliquots, and frozen at −70°C. Whole blood samples for lymphocyte blastogenesis and flow cytometry were processed as described below.

#### Serology

For both experiments, pre- and post-vaccination serum samples were sent to the National Veterinary Services Laboratory for brucellosis testing by standard card (CARD), standard plate (PLATE), rivanol (RIV), buffered acidified plate antigen (BAPA), complement fixation (CF), and fluorescence polarization assay (FPA) (Alton et al., 1988; Nielsen et al., 2000; Rhyan et al., 2009; Schumaker et al., 2010). In both experiments pre- and post-vaccination, antibody titers at various time points against sRB51 were determined by a previously described ELISA procedure using gamma-irradiated sRB51 (Olsen et al., 1997, 2006) and a peroxidase-conjugated, mouse anti-goat IgG (heavy and light chain-specific) used at a 1:3000 dilution (Jackson Immunoresearch, West Grove, PA).

In both experiments, serum collected just prior to and after experimental challenge with s2308 was evaluated using the standard tube agglutination test (STAT) (Alton et al., 1988).

#### Lymphocyte blastogenesis

At 6 and 10 wk post-vaccination (Experiment 1 only), and 6 and 14 wk post-challenge, whole blood was collected in an acid-citrate dextrose solution and peripheral blood mononuclear cells (PBMC) were prepared for lymphocyte blastogenesis and cell proliferation assays, as described by Olsen et al. (2009). Fifty microliter of PBS containing 5 × 10^5^ cells, were added to flat-bottom wells of 96-well microtiter plates that contained gamma-irradiated s19 or sRB51 (10^9^–10^5^ bacteria/well). Wells containing no antigen served as negative controls and wells containing 1 μg/ml pokeweed mitogen (Sigma Chemical Corp, St. Louis, Missouri, USA) were used as positive controls for proliferative responses. Cell proliferation results were converted to stimulation indices (counts per minute [cpm] of wells containing antigen/cpm in the absence of antigen) for statistical comparisons.

#### Flow cytometry

In Experiment 1 only, at 6 and 14 wk post-challenge, aliquots of PBMC containing 2 × 10^7^ cells were prepared for and analyzed through flow cytometry as previously described (Olsen et al., 2009). Primary antibodies (VMRD, Pullman, WA) included anti-CD4 (17D1-immunoglobulin G [IgG]), anti-CD8 (ST8-IgM), anti-B cell (PIG45AIgG2b), anti-γδTCR (GB21A-IgG2b), and anti-WC1 (BAQ4A-IgG1). Secondary antibodies included peridinin chlorophyll protein (1 μg/ml)-conjugated rat anti-mouse IgG1 (Becton Dickinson, Franklin Lakes, NJ, USA) and phycoerythrin (1 μg/well)-conjugated goat anti-mouse IgM or IgG2b (Southern Biotechnology Associates, Birmingham, AL). Data were analyzed using commercially available software: (CellQuest; Becton Dickinson and Modfit;Verity Software House, Inc., Topsham, ME, USA).

#### Necropsy and tissue collection

Twenty-four hr after parturition, cows were anesthetized and cow and calf euthanized by intravenous administration of sodium pentobarbital (88 mg/kg; Fort Dodge Laboratories, Ft. Dodge, Iowa, USA). Calves were considered viable if it was observed to stand and showed evidence of having nursed (observed/milk in stomach). Maternal samples obtained at necropsy included: lung, liver, spleen, placentome/caruncle, mammary gland tissue, and milk from all four quarters, lymphatic tissue (bronchial, hepatic, internal iliac, mandibular, mesenteric, parotid, retropharyngeal, prescapular, and supramammary), blood, vaginal swab, and conjunctival swab. Fetal samples obtained included: lung, liver, spleen, bronchial lymph node, gastric contents, rectal swab, and blood.

#### Brucella culture and histopathologic preparation

For *Brucella* culture, blood, other fluids, and swab samples were inoculated directly on tryptose agar plates containing 5% bovine serum. Tissue samples were triturated in 0.85% NaCl using a tissue grinder, and placed on tryptose agar plates containing 5% bovine serum. After incubation at 37°C and 5% CO_2_ for 7 days, *B. abortus* was identified by colony morphology and growth characteristics and confirmed by a *Brucella-*specific polymerase chain reaction assay (Alton et al., 1988; Lee et al., 2001). For histopathology, tissues were placed in neutral buffered 10% formalin, embedded in paraffin, sectioned at a thickness of 5 μm, and stained with hematoxylin and eosin.

### Data analysis

Serologic data obtained from ELISA, STAT, and data obtained from proliferation assays were analyzed as the logarithm of the value. STAT data that were negative at a 1:25 dilution were converted to 1 prior to conversion to a log value. Data from ELISA and STAT assays were compared over all time points using a Two-way analysis of variance model (ANOVA), whereas differences between groups in flow cytometry and [^3^H]thymidine incorporation (lymphocyte blastogenesis) at each time-point were compared by a general linear model procedure (SAS Institute, Inc., Cary, NC). Means for individual treatments were separated by use of a least-significant-difference procedure (*P* < 0.05). Numbers of culture positive tissues were compared among groups using Fisher's Exact Test (SAS Institute Inc.; *P* < 0.05).

## Results

### Serology

Serologic data were obtained from the elk in order to evaluate reaction to standard tests and sRB51 antigen after vaccination with sRB51+*SOD*c, *Wbo*A, as well as extent of antibody responses to s2308 antigen in the face of *B. abortus* challenge.

#### Experiment 1

**Figure 1:**
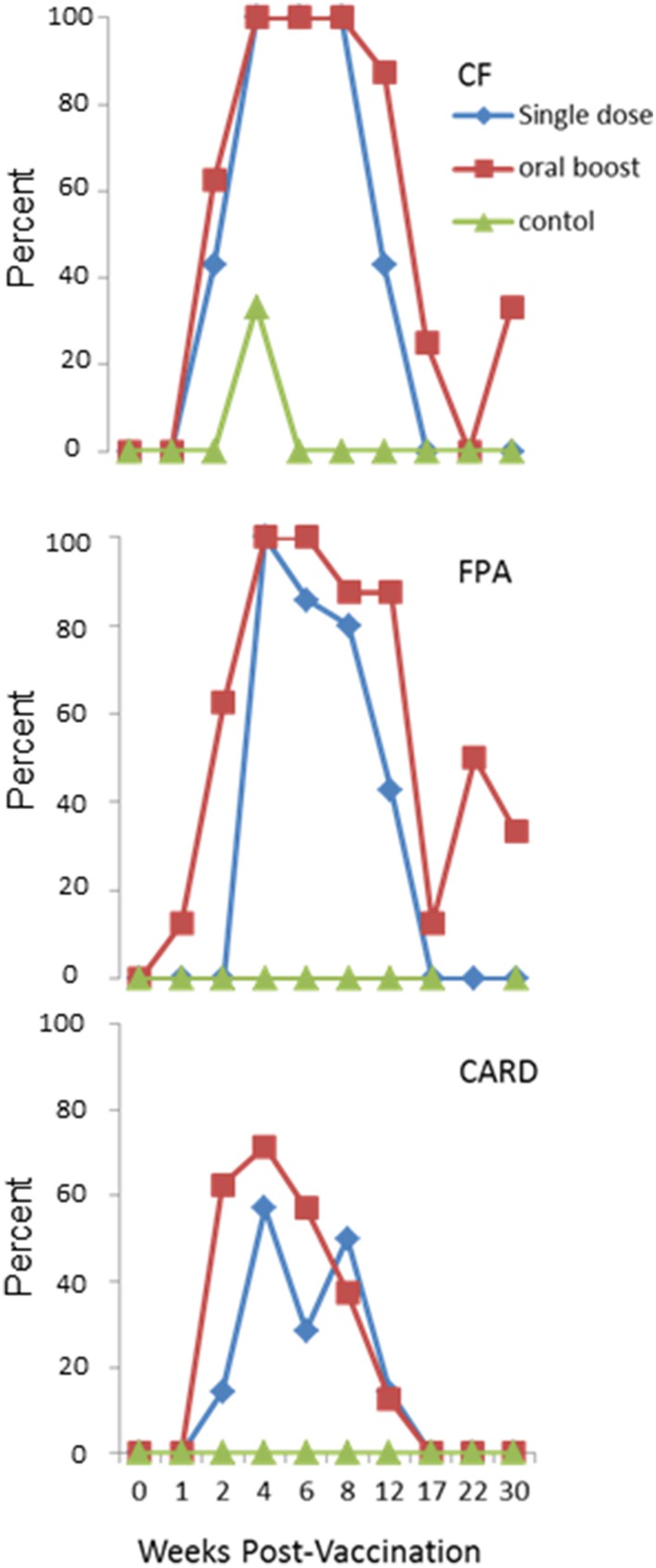
**Experiment 1: Percent positive elk in each vaccine group as measured on three standard brucellosis tests at multiple time points: Complement Fixation Assay (CF), Fluorescence Polarization Assay (FPA), and Standard Card Test (CARD)**. Animals in both vaccination groups developed antibodies that caused positive responses at or after 2 wk post-prime vaccination on all three tests. Positive responses on CF declined after 8 wk post-prime in both vaccine groups. Positive responses to FPA decreased after 4 wk post-prime vaccination in the single dose group and after 6 wk post-prime vaccination in the oral boost group. Positive responses on the CARD test declined after 4 wk post-prime vaccination in both groups. Elk in the single dose and oral boost groups were vaccinated with 2 × 10^10^ colony forming units sRB51+*SOD*c, *Wbo*A intramuscularly (0 wk) and elk in the oral boost group were orally vaccinated with 1 × 10^11^ colony forming units sRB51+*SOD*c, *Wbo*A at wk 6. The control group received phosphate buffered saline intramuscularly.

All elk were negative on CARD, CF, and FPA tests prior to vaccination. Transient positive responses on these serologic tests occurred in both vaccine groups after vaccination. Animals in both vaccination groups developed humoral responses that caused positive responses on the CARD, CF, and FPA tests at or after 2 wk post-prime vaccination, indicating response to the overexpressed antigens in the vaccine. For the CF, positive responses declined after 8 wk post-prime in both vaccine groups, with a greater rate of decline in the single dose treatment as compared to the oral boost treatment. For the FPA, positive responses decreased after 4 wk post-prime vaccination in the single dose group and in the oral boost group after 6 wk post-prime vaccination. Positive responses on the CARD test declined after 4 wk post-initial vaccination in both groups. Data are represented in Figure 1. Most animals in all three treatment groups were positive on PLATE, RIV, and BAPA prior to initial vaccination (data not shown). These data indicate that parenteral vaccination with sRB51+*SOD*c, *Wbo*A induces antibody production detectable on standard *Brucella* tests for up to 8 wk, and that antibody levels in the oral boost group lasted somewhat longer. The reason for the pre-vaccination positive tests outcomes (PLATE, RIV, and BAPA) is unknown and is addressed further in the discussion.

**Figure 2:**
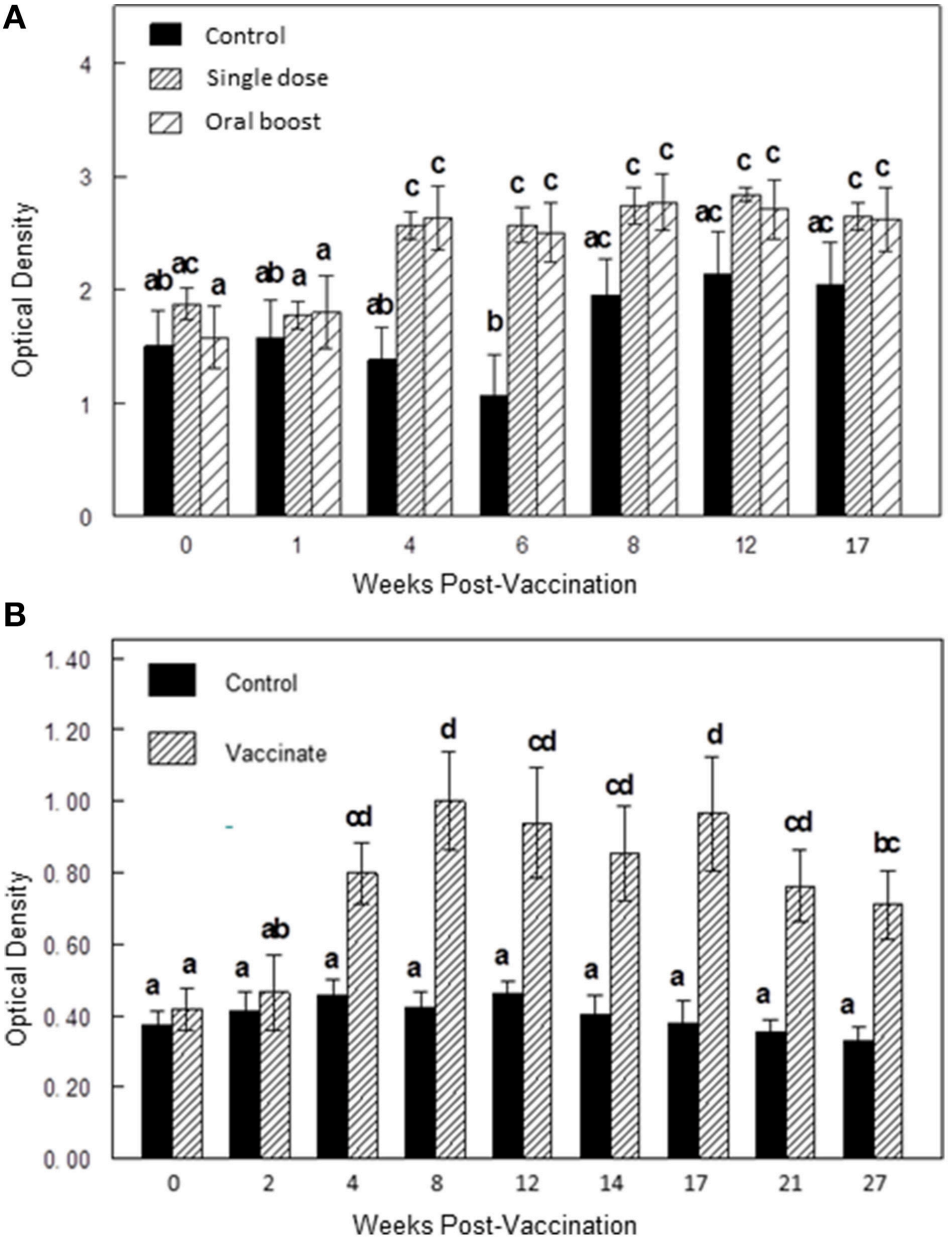
**(A,B)** Differences in antibody responses (ELISA; mean optical density ± SEM) to sRB51 between vaccination groups at multiple time points in Experiment 1 **(A)** and Experiment 2 **(B)**. **(A)** In Experiment 1, mean responses to sRB51 were greater (*P* < 0.05) in the single dose group and the oral boost group at 4 and 6 wk after initial vaccination as compared to serologic responses of the control group. Elk in the single dose and oral boost groups were vaccinated with 2 × 10^10^ colony forming units sRB51+*SOD*c, *Wbo*A intramuscularly (0 wk) and elk in the oral boost group were orally vaccinated with 1 × 10^11^ colony forming units sRB51+*SOD*c, *Wbo*A at wk 6. The control group received phosphate buffered saline intramuscularly. **(B)** In Experiment 2, mean responses to the sRB51 were greater (*P* < 0.05) in the vaccinated group at 4, 8, 12, 14, 17, 21, and 27 wk after initial vaccination as compared to controls. Elk in the vaccinate group were orally vaccinated with 1 × 10^11^ colony forming units sRB51+*SOD*c, *Wbo*A at wk 0. Elk were boosted with the same preparation at wk 40 although this figure does not reflect antibody responses post-booster. The control group received diluent orally. In both figures, bars with differing letters differ significantly from each other (*p* < 0.05).

Mean ELISA responses to sRB51 were greater (*P* < 0.05) in the single dose group and the oral boost group at 4 and 6 wk after initial vaccination as compared to serologic responses of the control group indicating antibody production against sRB51 after vaccination (Figure 2A).

Prior to experimental challenge with s2308, mean titers on the STAT did not differ (*P* > 0.05) between groups. Both control and single dose groups demonstrated seroconversion, indicating significant antibody production in the face of *B. abortus* infection, in that mean titers at 6 wk after challenge were greater (*P* < 0.05) when compared to serologic data obtained prior to challenge. Mean STAT titer of oral boost vaccinates after experimental challenge did not differ (*P* > 0.05) from the mean titer prior to challenge. Control animals had greater (*P* < 0.05) mean STAT titers at 6 and 14 wk post-challenge, and at necropsy, when compared to response of animals in oral boost group and at 14 wk and necropsy when compared to the single dose group.

Mean titers of oral boost and single dose groups were significantly different from each other at 6 wk post-challenge (Two-way ANOVA; *p* < 0.05) but did not differ (*P* > 0.05) at any other sampling time. Increased antibody responses after *B. abortus* challenge are indicative of response to infection. Although, it is not clear what the differences in responses among the vaccine groups and controls indicate, it may be that the oral boost group had reduced antibody responses to infection via the mucosa route in the face of mucosal delivery of the booster vaccine (see Table 1).

**Table 1:** **Elk Serum Tube Agglutination Test titers (± SEM) at various time points after challenge with 10^7^ cfu *Brucella abortus* s2308 and at necropsy**.

	**Day 0**	**4 wk PC**	**6 wk PC**	**8 wk PC**	**12 wk PC**	**14 wk PC**	**Necropsy**	**Calf**
**EXPERIMENT 1**								
Single Dose	4 ± 4^a^	ND	29 ± 15^b^	ND	ND	8 ± 5^a^^c^	4 ± 4^a^	4 ± 4^a^
Oral boost	0 ± 0^a^	ND	12 ± 8^a^^c^	ND	ND	8 ± 8^a^	0 ± 0^a^	0 ± 0^a^
Control	0 ± 0^a^	ND	133 ± 54^b^	ND	ND	58 ± 14^b^	21 ± 8^c^	6 ± 6^a^^c^
**EXPERIMENT 2**								
Oral	0 ± 0^a^	35 ± 12^c^	ND	20 ± 7^c^	5 ± 3^a^	ND	5 ± 3^a^	0 ± 0^a^
Control	0 ± 0^a^	146 ± 46^b^	ND	76 ± 21^b^	25 ± 8^c^	ND	4 ± 4^a^	4 ± 4^a^

Values superscripted with different letters within experiments are significantly different from each other. wk, weeks.

ND, not done; PC, post-challenge.

#### Experiment 2

With the exception of one vaccinated elk that was positive on the PLATE and one control that was positive on the CARD, the remaining elk were negative on all serologic tests prior to initial vaccination. Pre-vaccine serology was not performed on five elk in the vaccination treatment due to accidental loss of samples. At 3 wk post-vaccination, seven of 13 vaccinates tested were positive on PLATE and one animal was positive on BAPA, FPA, and CARD as well. Three of 15 control animals tested at this time point were also positive on PLATE. Animals were sporadically positive on BAPA, PLATE, and FPA after booster vaccination; however these results were isolated and no positive responses were detected over two or more consecutive time points (data not shown). These results indicate that oral sRB51+*SOD*c, *Wbo*A vaccination induces transient antibody production detectable on standard *Brucella* tests.

Mean ELISA responses to the sRB51 were greater (Two-way ANOVA; *P* < 0.05) in the vaccinated group at 4, 8, 12, 14, 17, 21, and 27 wk after initial vaccination as compared to controls (Figure 2B).

Mean STAT titers did not differ (*P* > 0.05) between control and oral vaccination groups prior to challenge. Similar to the first experiment, both the vaccine and control groups demonstrated greater (*P* < 0.05) mean STAT titers at 4 and 8 wk after challenge when compared to mean titer of sera obtained prior to challenge. Also, similar to responses in Experiment 1 after challenge, mean titers of the control group were greater than those of vaccinates (*P* < 0.05) at 4, 8, and 12 wk after challenge, but responses did not differ significantly at necropsy. Again, it unclear what these differences in antibody responses indicate, but they may be associated with responses to mucosal vaccination followed by mucosal *B. abortus* challenge (see Table 1).

### Lymphocyte blastogenesis

#### Experiment 1

Lymphocyte proliferative responses of PBMC to irradiated sRB51 were greater (*P* < 0.05) in the oral boost group at 6 (4.4-fold difference) and 10 wk (12.6-fold difference) after initial vaccination when compared to mean responses of PBMC from non-vaccinated elk. Mean responses of PBMC from the single dose group did not differ (*P* > 0.05) at 6 and 10 wk from responses of elk in the oral or control treatment groups. At 14 wk after challenge PBMC from the oral boost group had greater mean proliferative response as compared to the control group (28.3-fold difference; *P* < 0.05). Mean proliferative responses of PBMC obtained from elk in the single dose group at 6 and 14 wk and from the oral group at 6 wk after challenge differed, but not significantly (*P* > 0.05), to mean responses in the control group (data not shown). These results indicate evidence of increased cellular immune response to sRB51 antigen in the face of vaccination in the oral boost group as well as at 14 wk after challenge as compared to non-vaccinated animals.

### Flow cytometry

#### Experiment 1

At 6 wk post-challenge, there were no significant differences among the three groups regarding proliferative responses to killed sRB51 by any of the cell types evaluated. At 14 wk post-challenge PBMC obtained from the oral boost group produced greater (*P* < 0.05) total cell proliferation than PBMC obtained from the single dose or control groups (Table 2). This is consistent with the increased cellular immune responses seen in the lymphocyte blastogenesis data obtained from the oral boost group post-challenge. Analysis did not identify any other differences in flow cytometric data.

**Table 2:** **Experiment 1: Mean flow cytometric responses (± SEM) to sRB51 after challenge with 10^7^ cfu *B. abortus* s2308**.

**Time after challenge**	**Vaccine Group^b^**	**Cell type**				
		**Total^a^**	**CD4+**	**CD8+**	**γδTCR+**	**IgM+**
6 wk	Single dose	55 ± 462	0 ± 0	778 ± 939	149 ± 227	388 ± 499
	Oral boost	895 ± 1503	58 ± 297	404 ± 571	732 ± 762	388 ± 499
	Control	0 ± 0	0 ± 0	61 ± 785	55 ± 48	109 ± 134
14 wk	Single dose	191 ± 383	0 ± 0	0 ± 0	767 ± 556	332 ± 316
	Oral boost	3002 ± 1344^c^	330 ± 681	1372 ± 904	1307 ± 680	117 ± 199
	Control	111 ± 431	0 ± 0	250 ± 214	450 ± 214	62 ± 51

a Mean proliferating cells per 10,000 PBMC ± standard deviation.

b n = 5/Vaccine Group.

c Significantly different from other two groups.

### Parturition and necropsy

#### Calves

Numbers of abortions were documented in both experiments in order to evaluate the efficacy of sRB51+*SOD*c, *Wbo*A in preventing abortions after infection.

##### Experiment 1

All animals in the single dose group (*n* = 6) and all animals in the oral boost group (*n* = 6) gave birth to live calves; however one calf in the single dose group was not considered viable based on clinical assessment and lack of milk in its abomasum. In the control group, two of six cows gave birth to dead calves and one calf was considered not viable. There were no significant differences among the three groups regarding numbers of abortions (Fisher's Exact Test; *p* ≥ 0.05) or numbers of abortions and non-viable calves combined (*p* > 0.05; see Table 3).

**Table 3:** **Percentage and number of abortions or animals with specific tissue types positive for *B. abortus* s2308 in each group**.

**Vaccine Group**	**Abortions^a^**	**Fetal tissues^a^^,^^b^**	**Repro/Mammary tissues^a^^,^^c^**	**Other tissues^a^^,^^d^**	**Any tissue**
**EXPERIMENT 1**					
Single dose	0 (0/6)	17 (1/6)	33 (2/6)	33 (2/6)	33 (2/6)
Oral boost	0 (0/6)	17 (1/6)	17 (1/6)	50 (3/6)	50 (3/6)
Control	33 (2/6)	17 (1/6)	67 (4/6)	83 (5/6)	83 (5/6)
**EXPERIMENT 2**					
Oral vaccinate	0 (0/10)^e^	40 (4/10)^e^	10 (1/10)^e^	64 (7/11)	82 (9/11)
Control	0 (0/7)	57 (4/7)	29 (2/7)	57 (4/7)	100 (7/7)

a % (Number of aborted or infected/total).

b Fetal Tissues included lung, liver, spleen, bronchial lymph node (ln), gastric contents, cerebral spinal fluid, rectal swab, blood.

c Reproductive (Repro)/mammary tissues included milk, mammary gland, vaginal swab, supramammary ln, placentome.

d Other Tissues included lung, liver, spleen, lymphatic tissue (bronchial, hepatic, internal iliac, mandibular, mesenteric, parotid, retropharyngeal, and prescapular), blood, and conjunctival swab.

e One vaccinated elk not pregnant and therefore not included in this category.

##### Experiment 2

All of the animals except one non-pregnant vaccinate gave birth to live calves. No abortions or non-viable calves were observed.

#### Culture

Culture data were obtained from various tissues in both experiments in order to evaluate the efficacy of sRB51+*SOD*c, *Wbo*A in preventing or reducing colonization of these tissues in the face of *B. abortus* infection.

##### Experiment 1

*B. abortus* was recovered at necropsy from at least one tissue in five vaccinated elk, with two of six elk culture positive in the single dose and three of six in the oral boost group. *Brucella abortus* was recovered from tissue samples of five of six controls. Two of six animals in the single dose group and one of six in the oral boost group were culture positive in reproductive/mammary tissues (supramammary lymph node, milk, mammary gland, placentome/caruncle, and/or vaginal swab). In comparison, four of the six controls were culture positive for reproductive/mammary tissues. Groups did not differ (*P* > 0.05) when number of animals culture positive for *Brucella* were compared or when reproductive, fetal, or other maternal tissue groups were compared (Fisher's Exact Test; *p* ranging from 0.35 to 1.0). Two of 12 calves in vaccination groups (one each for single dose and oral boost groups) were culture positive. *Brucella* was recovered from one of six calves in the control group. This culture-positive fetus was one of the two abortions that occurred. The other aborted fetus was negative on culture. The two calves considered non-viable were culture negative as well (see Table 3 and Table S1).

##### Experiment 2

Seven of 10 pregnant elk in the vaccination group were culture positive for *B. abortus* at necropsy from at least one tissue type with only one animal positive for recovery of *Brucella* from mammary or uterine tissues. In comparison, six of the seven elk in the control group were culture positive on at least one tissue type with *Brucella* recovered from mammary or uterine tissues of two of seven cows. Four of seven calves in the control group, and four of 10 calves in the vaccination group were positive for recovery of *B*. *abortus*. The vaccine group did not differ (*P* > 0.05) from the control treatment in regards to incidence of recovery of *Brucella*, either overall or in the fetal, reproductive, or maternal tissues (Fisher's Exact Test; *p* ranging from 0.4 to 1.0; see Table 3 and Table S2).

Although it appeared that *B. abortus* was recovered in more control animals after challenge than in vaccinates in the first experiment, these differences were not significant. Therefore, neither experiment indicated a reduction of tissue colonization by *B. abortus* due to sRB51+*SOD*c, *Wbo*A vaccination.

#### Histopathology

##### Experiment 1

Microscopic lesions were identified in the mammary glands, uterus, liver, and placenta of the cow, and lung and liver of the fetus. Mammary glands of two of six cows in the single dose treatment, one of six in the oral boost treatment, and no cows in the control treatment displayed mild to moderate, focal, lymphoplasmacytic, or suppurative interstitial mastitis. Uterine lesions including mild to moderate endometritis with mixed inflammatory cell infiltrates were detected in five of six elk in the single dose treatment, four of six elk in the oral boost treatment, and two of six controls. Maternal carunculitis was seen in four of six elk in the single dose group, four of six in the oral boost group, and in two of six controls. Severity of carunculitis ranged from mild to marked with degenerate cells and neutrophil infiltrates. In some elk, focal hypercellularity in the interstitium of the caruncles was observed, composed of large mononuclear cells and focal accumulations of lymphocytes, plasma cells, and scattered neutrophils. One of the six elk in the oral boost group had multifocal, random, lymphoplasmacytic, and suppurative hepatitis, with occasional hepatocellular necrosis. Two calves from the single dose group had lymphoplasmacytic and suppurative interstitial pneumonia. Moderate to marked, multifocal, suppurative hepatitis with hepatocellular necrosis was also observed in one of these calves. Only one of the elk (control group) had histopathologic lesions that were associated with a positive tissue culture (see Table S1).

##### Experiment 2

Mammary glands of all vaccinated and six of seven control elk displayed marked glandular atrophy and degeneration, with dilation and inspissation of secretory material, surrounded by mixed chronic interstitial inflammation. With the exception of one cow, mild to marked, suppurative splenitis with lymphoid hyperplasia were observed in all elk but severity did not appear to differ between groups. Submandibular lymph nodes of all controls and six of nine vaccinated elk displayed mild to moderate suppurative lymphadenitis with lymphoid hyperplasia. Mild to marked diffuse suppurative placentitis was observed in six of seven controls and 10 of 10 vaccinates. Fetal lungs from four of seven controls and seven of 10 vaccinates displayed mild to moderate suppurative interstitial pneumonia. Mastitis, lymphadenitis, and placentitis lesions were slightly more severe in vaccinates as compared to controls. Recovery of *Brucella* did not appear to be associated with presence or severity of lesions (see Table S2).

## Discussion

Data from our two controlled efficacy studies suggest that parenterally and/or orally administered sRB51 + *SOD*c, *wbo*A did not induce significant levels of protection in elk against experimental *B. abortus* challenge. Based on encouraging data observed in mice (Vemulapalli et al., 2000a,b, 2002), we anticipated that mucosal vaccination with an RB51 strain that overexpresses both superoxide dismutase and glycosyltransferase, antigens that are immunogenic and associated with virulence, would elicit a protective response in elk. Elzer and Davis (1997) reported observing partial protection against abortion in elk orally vaccinated with 10^10^ cfu sRB51 and challenged 2 mo after inoculation. However, their results were in contrast to subsequent studies in elk performed by Cook et al. (2002) and Kreeger et al. (2002) where abortions and infection were not prevented by vaccination with parenteral sRB51 (hand injection and biobullet) when compared to non-vaccinated controls. In addition, in a controlled study in bison, parenteral sRB51 + *SODc, wboA* offered even less protection than the parenteral sRB51 strain, which was effective in preventing abortion and reducing infection in bison (Olsen et al., 2009, 2015).

Our conclusion regarding the lack of efficacy of sRB51 + *SODc, wboA* in elk was based on tissue culture alone, as we did not induce significant numbers of abortions in non-vaccinates after experimental challenge. A lack of efficacy of the vaccine strain is also supported by culture and histopathology data which demonstrated infection and histologic lesions without significant differences in these parameters between vaccinates and controls. We were surprised that high numbers of abortions were not observed since high numbers of elk in both groups were culture positive for the challenge strain. It remains unclear as to why experimental challenge using the standard ruminant challenge model (1 × 10^7^ cfu s2308 conjunctivally in mid-gestation) did not induce high numbers of abortions, particularly in control elk. Cook et al. (2002) and Kreeger et al. (2002) successfully induced abortions in the majority of their elk using a similar dose of s2308, and the same inoculum as was used in this study effectively caused abortions in a parallel experiment in bison (Olsen et al., 2015). Similarly prepared challenge inocula induced significant numbers of abortion in non-vaccinated cattle (Olsen and Johnson, 2011). Data have demonstrated immunologic differences between elk and cattle in responses to tuberculosis and brucellosis vaccines (Waters et al., 2003; Olsen et al., 2006), which may alter the pathologic mechanisms associated with induction of abortion. One example might include the observation that serum tube agglutination antibody titers were generally lower than those observed in bison and cattle after experimental challenge (Olsen and Johnson, 2011). Our data suggest a need for further studies to establish a more consistent and reliable model for evaluation of vaccine efficacy against brucellosis in elk that mimics pathologic effects under field conditions.

It is interesting that lesions of placentitis and fetal interstitial pneumonia commonly associated with *B. abortus* infection were found in the challenged elk and their calves from which the challenge strain was not recovered. It cannot be eliminated that these lesions may reflect *Brucella* infection despite the failure to recover the challenge strain from the tissue samples. Alternatively, these lesions may have represented other pathologic conditions that we cannot diagnose at this time.

Although a majority of vaccinated elk in the first experiment were serologically positive prior to vaccination, these seropositive responses were in three *Brucella* tests used for screening. Screening tests are known for having high sensitivity but relatively lower specificity. As these animals originated from a brucellosis-free captive herd in a brucellosis-free state (Minnesota, USA), we believe it is highly unlikely they had been exposed to brucellosis. It is far more likely that the animals developed antibodies after infection with one of the various organisms that are known to cause cross-reactions on brucellosis serologic tests. *Yersinia enterocolitica* O:9 is one example as it possesses an identical lipopolysacharide to *B. abortus* and has been reported in numerous studies as a cause of false positive serologic responses on brucellosis tests (Weynants et al., 1996; Garin-Bastuji et al., 1999). However, as other bacteria have also been identified as being capable of causing cross-reactors in brucellosis serologic tests, it cannot be excluded that they may have been responsible for the false-positive responses in the current study (Nielsen et al., 2004).

Current regulations require that *Brucella*-infected animals be held under ABSL-3 conditions which is expensive and to some extent limits experimental units in a study. We cannot exclude the possibility that statistical differences in infection or abortions between experimental groups may have been detected if greater sample sizes were evaluated. However, numbers of the animals in the current experiment should have been sufficient to identify a protective vaccine that would be suitable for use under field conditions. As delivery of vaccine to a free-ranging population will most likely be expensive and complicated, only those vaccines that demonstrate high levels of protection under controlled experiments are likely candidates for consideration of further evaluation under field conditions. Although sRB51 + *SOD*c, *wbo*A did appear to slightly reduce infection in the two studies, as compared to controls, this effect was marginal in preventing fetal, mammary, or uterine infection. Therefore, the data suggest its effect on disease prevalence under field conditions would be limited, and therefore further evaluation of this vaccine in elk is not warranted.

Development of a highly efficacious vaccine that protects elk against *B. abortus* infection has yet to be achieved. Continuation of efforts to develop a brucellosis vaccine in elk is important, as elk have been implicated as the source of *Brucella* infection in a number of cattle herds in the GYA and vaccination could serve as a vital component for reducing disease prevalence in elk. Future studies not only need to contribute to development of a more protective brucellosis vaccine for elk, but should also improve the *Brucella* challenge model for elk such that it mimics clinical characteristics of the disease under field conditions. Development of a protective vaccine that can be effectively delivered to free-ranging elk populations would be a valuable resource for agencies with responsibilities related to brucellosis control or management of elk in the GYA.

## Author contributions

PN, SO, JR, NS, MM, PS, RB, MS participated in design of study. PN, SO, JR, NS, MM, SH, AP, PS, RB participated in execution of study and analysis of samples and data. PN, SO, JR, NS, MM, SH, SP, PS, RB, SB, MS participated in writing of manuscript.

## Funding

This research was funded by the United States Department of Agriculture.

### Conflict of interest statement

The authors declare that the research was conducted in the absence of any commercial or financial relationships that could be construed as a potential conflict of interest.
