# Supplementary material for: Vaccination of Elk (Cervus canadensis) with Brucella abortus Strain RB51 Overexpressing Superoxide Dismutase and Glycosyltransferase Genes Does Not Induce Adequate Protection against Experimental Brucella abortus Challenge
Source: Front Cell Infect Microbiol. 2016 Feb 10;6:10. doi: 10.3389/fcimb.2016.00010 (PMC4748031; doi:10.3389/fcimb.2016.00010)
Supplement: Supplementary file 1 [file Table1.DOCX]

Table S1. Experiment 1: Calf status, number of culture positive tissues, and outcome of histopathological tissue examination

corresponding to individual elk

|  |  |  | Tissue Culture Status^a^ | | |  |
| --- | --- | --- | --- | --- | --- | --- |
| Animal ID | Vaccine Group | Calf Status | Fetal Tissues^b^ | Cow Repro/Mammary Tissues^b^ | Cow Other Tissues^b^ | Histopathology^f^ |
| 28 | Single Dose | Live | 1/8 | 2/5 | 9/13 | carunculitis; endometritis |
| 43 | Single Dose | Live | 0/8 | 0/5 | 0/13 | endometritis |
| 89 | Single Dose | Live | 0/8 | 0/5 | 0/13 | mastitis; carunculitis; endometritis |
| 107 | Single Dose | Live | 0/8 | 2/5 | 1/13 | NSL |
| 726 | Single Dose | Live | 0/8 | 0/5 | 0/13 | carunculitis; endometritis; fetal pneumonia |
| 936 | Single Dose | Live^d^ | 0/8 | 0/5 | 0/13 | carunculitis; endometritis; fetal pneumonia/hepatitis |
|  |  |  |  |  |  |  |
| 1 | Oral Boost | Live | 0/8 | 1/5 | 1/13 | mastitis |
| 116 | Oral Boost | Live | 0/8 | 0/5 | 0/13 | NSL |
| 125 | Oral Boost | Live | 1/8 | 0/5 | 1/13 | carunculitis; endometritis |
| 304 | Oral Boost | Live^c^ | 0/8 | 0/5 | 2/13 | carunculitis; endometritis |
| 353 | Oral Boost | Live | 0/8 | 0/5 | 0/13 | carunculitis; endometritis; hepatitis |
| 355 | Oral Boost | Live | 0/8 | 0/5 | 0/13 | carunculitis; endometritis |
|  |  |  |  |  |  |  |
| 32 | Control | Live^d^ | 0/8 | 0/5 | 0/13 | NSL |
| 55 | Control | NA | 0/8 | 2/5 | 2/13 | NSL |
| 115 | Control | Live^e^ | 0/8 | 2/5 | 0/13 | NSL |
| 364 | Control | Dead | 0/8 | 0/5 | 1/13 | NSL |
| 365 | Control | Live^d^ | 0/8 | 1/5 | 1/13 | carunculitis; endometritis |
| 949 | Control | Dead | 7/8 | 5/5 | 10/13 | carunculitis; endometritis^g^ |

^a^ Positive/Total number of tissues tested

^b^ Fetal tissues include: lung, liver, spleen, bronchial lymph node (ln), gastric contents, cerebral spinal fluid, rectal swab, blood; Cow repro/mammary tissues include: milk, mammary gland vaginal swab, supramammary ln, placentome; Cow other tissues include: lung, liver, spleen, bronchial ln, hepatic ln, internal iliac ln, mandibular ln, mesenteric ln, parotid ln, retropharyngeal ln, blood, conjunctival swab, prescapular ln.

^c^ Cow had no milk. No milk in calf stomach. SI dilated with gas suggesting nursing. Caretakers thought it nursed.

^d^ Calves that probably were not viable. No milk in stomach and/or lack of ambulatory function. Calf 936 killed by dam.

^e^ No milk in calf stomach. Was walking.

^f^NSL: no significant lesions

^g^ Elk was culture positive on placentome
