# Supplementary material for: Vaccination of Elk (Cervus canadensis) with Brucella abortus Strain RB51 Overexpressing Superoxide Dismutase and Glycosyltransferase Genes Does Not Induce Adequate Protection against Experimental Brucella abortus Challenge
Source: Front Cell Infect Microbiol. 2016 Feb 10;6:10. doi: 10.3389/fcimb.2016.00010 (PMC4748031; doi:10.3389/fcimb.2016.00010)
Supplement: Supplementary file 2 [file Table2.docx]

Table S2. Experiment 2: Calf status, number of culture positive tissues, and outcome of histopathological tissue examination corresponding to individual elk

|  |  |  | Tissue Culture Status^a^ | | |  |
| --- | --- | --- | --- | --- | --- | --- |
| Animal ID | Vaccine Group | Calf Status | Fetal Tissues^b^ | Cow Repro/Mammary Tissues^b^ | Cow Other Tissues^b^ | Histopathology |
| 4 | Oral Vx | Live | Neg | Neg | 2/13 | mastitis-marked; carunculitis-mild; splenitis-marked |
| 7 | Oral Vx | Live | 1/8 | Neg | Neg | mastitis-marked; carunculitis-min; splenitis-mod |
| 8 | Oral Vx | Live | 1/8 | Neg | Neg | mastitis-marked; carunculitis-min; splenitis-mod |
| 10 | Oral Vx | Live | 1/8 | Neg | 1/13 | mastitis-min; carunculitis-mild; splenitis-mild |
| 21 | Oral Vx | Live | Neg | Neg | Neg | Mastitis-mild; carunculitis-mild; splenitis-mod |
| 23 | Oral Vx | Live | 1/8 | Neg | 1/13 | Mastitis-mild; carunculitis-mild; splenitis-mod |
| 26 | Oral Vx | Live | Neg | Neg | 1/13 | Mastitis-mild; carunculitis-mod; splenitis-min |
| 29 | Oral Vx | Live | Neg | 1/5 | 4/13 | Mastitis-mod; splenitis-mild |
| 30 | Oral Vx | Live | Neg | Neg | 1/13 | Mastitis-mild; carunculitis-mild; splenitis-mild |
| 31 | Oral Vx | Live | Neg | Neg | 1/13 | Mastitis-mild; carunculitis-mild; splenitis-min |
| 36 | Oral Vx | NA | NA | Neg | Neg | Mastitis-min |
|  |  |  |  |  |  |  |
| 9 | Control | Live | Neg | Neg | 2/13 | Mastitis-mild: carunculitis-mild; splenitis-mild |
| 15 | Control | Live | 1/8 | Neg | 2/13 | Mastitis-mild: carunculitis-mild; splenitis-mod |
| 16 | Control | Live | 1/8 | Neg | 1/13 | Mastitis-min; splenitis-mild |
| 22 | Control | Live | Neg | 1/5 | Neg | Mastitis-mild; carunculitis-marked; splenitis-mild |
| 28 | Control | Live | 2/8 | 1/5 | Neg | carunculitis-min; splenitis-mild |
| 32 | Control | Live | Neg | Neg | 1/13 | Mastitis; carunculitis-min; splenitis-mod |
| 33 | Control | Live | 2/8 | Neg | Neg | Mastitis-mod; splenitis-mod |

^a^ Postive/Total number of tissues tested

^b^ Fetal tissues include: lung, liver, spleen, bronchial lymph node (ln), gastric contents, cerebral spinal fluid, rectal swab, blood; Cow repro/mammary tissues include: milk, mammary gland vaginal swab, supramammary ln, placentome; Cow other tissues include: lung, liver, spleen, bronchial ln, hepatic ln, internal iliac ln, mandibular ln, mesenteric ln, parotid ln, retropharyngeal ln, blood, conjunctival swab, prescapular ln.
